# Beechwood carbohydrates for enzymatic synthesis of sustainable glycolipids

**DOI:** 10.1186/s40643-017-0155-7

**Published:** 2017-06-07

**Authors:** Sascha Siebenhaller, Tatjana Hajek, Claudia Muhle-Goll, Miriam Himmelsbach, Burkhard Luy, Frank Kirschhöfer, Gerald Brenner-Weiß, Thomas Hahn, Susanne Zibek, Christoph Syldatk

**Affiliations:** 1 Institute of Process Engineering in Life Sciences, Section II: Technical Biology, Karlsruhe Institute of Technology (KIT), Engler-Bunte-Ring 3, 76131 Karlsruhe, Germany; 2 Institute of Organic Chemistry and Institute for Biological Interfaces 4, Karlsruhe Institute of Technology, Karlsruhe, Germany; 3 Institute of Functional Interfaces, Karlsruhe Institute of Technology, Karlsruhe, Germany; 4 Fraunhofer Institute for Interfacial Engineering and Biotechnology, Stuttgart, Germany

**Keywords:** Deep eutectic solvents, Glycolipid synthesis, Lignocellulose, Lipase, Transesterification

## Abstract

Moving away from crude oil to renewable resources for the production of a wide range of compounds is a challenge for future generations. To overcome this, the use of lignocellulose as substrate can contribute to drastically reduce the consumption of crude oil. In this study, sugars from lignocellulose were used as a starting material for the enzymatic synthesis of surface-active sugar esters. The substrates were obtained by an acid-catalyzed, beechwood pretreatment process, which resulted in a fiber fraction that is subsequently hydrolyzed to obtain the monosaccharides. After purification and drying, this glucose- and xylose-rich fraction was used to create a deep eutectic solvent, which acts both as solvent and substrate for the lipase-catalyzed reaction at the same time. Finally, the successful synthesis of glycolipids from a sustainable resource was confirmed by ESI–Q–ToF mass spectrometry and multidimensional NMR experiments. Moreover, conversion yields of 4.8% were determined by LC–MS/MS.

**Electronic supplementary material:**

The online version of this article (doi:10.1186/s40643-017-0155-7) contains supplementary material, which is available to authorized users.

## Background

Currently, the world’s economy is running on crude oil, a limited resource. It is the starting product for fuels, plastics, chemicals, and pharmaceuticals; furthermore, it covers a large part of the world's energy supply. But the wastefulness of crude oil causes harm to the earth and the fear about the end of cheap oil is a widespread concern in people’s mind. This sets a tremendous focus on alternative resources for the production of more environmentally friendly fuels and chemicals.

An alternative resource and major contributor in the future will be biomass, with the main component being lignocellulose (Herrera 2006; Wyman 1999). As the major structural component of woody and non-woody plants, lignocellulose is the most abundant organic material in the world (Howard et al. 2003; Zhao et al. 2009). It is estimated that the worldwide production of lignocellulose ranges from 120 to 140 billion tons per year, which is over half of the world’s biomass (Pauly and Keegstra 2008). Lignocellulose is widely used for burning and heating but ultimately it is too valuable for that due to its high potential for material use. Lignocellulose has three major components: lignin, cellulose, and hemicelluloses, with carbohydrates accounting for up to 75% of the total amount (Jørgensen et al. 2007). Lignocellulose often occurs as garden waste or as residue in various branches of industry, so these sugars do not compete with the food or feed industry and do not have to be cultivated separately like corn or sugarcane. In order to utilize these huge quantities of sugars, a pretreatment is necessary because of the recalcitrance of the lignocellulose and to make the carbohydrates available for succeeding hydrolysis (Rubin et al. 2007). To achieve this, many processes were invented in the last decades to make the sugar accessible. One of them applied within the frame of this work is a chemical–physical process which uses an aqueous-organic solvent system under high pressure and temperature to separate the lignin from the carbohydrates (Kumar et al. 2009; Laure et al. 2014; Pan et al. 2008). The cellulose-rich fiber obtained is hydrolyzed and commonly applied as a potent substrate for microbial fermentation processes (Nigam 2001; Olsson and Hahn-Hägerdal 1996). Scientists currently do not focus on the enzymatic synthesis of sugar esters (glycolipids) which are surface-active agents although sustainable surfactants are highly demanded. The emulsifying properties and foaming ability of the glycolipids enable the application in cosmetics, pharmaceuticals, or the food industry (Banat et al. 2000; Marchant and Banat 2012; Šabeder et al. 2006). Furthermore, glycolipids are very mild biocompatible agents and skin-friendly, making them particularly suitable for the use in cosmetics (Chang and Shaw 2009; Pöhnlein et al. 2015). In addition, many glycolipids suppress bacterial growth so there are still several additional application possibilities (Šabeder et al. 2006). The synthesis of glycolipids from these sustainable sugars would have also the advantage that crude oil could be replaced by a renewable resource, since nowadays almost all surfactants are petroleum based (Cameotra and Makkar 1998).

The enzymatic synthesis of sugar esters can be performed using a lipase in a nearly water-free environment. Under these conditions, lipases reverse their hydrolytic activity and form ester bonds between a hydroxyl group of a sugar and the carboxyl group of a fatty acid (Klibanov 1989; Zaks and Klibanov 1985). A reason for the hitherto low industrial production of sugar esters by enzymes is the low solubility of sugars in non-polar organic solvents, in which lipases show their esterification activity. Vice versa, in more polar solvents in which sugars are soluble, most lipases lose their esterification activity (Plou et al. 2002). There are opportunities to increase the solubility of sugars, e.g., by supplementation with additional agents like dimethyl sulfoxide (DMSO), but this might interfere with the enzyme activity and stability (Castillo et al. 2003; Kitagawa et al. 1999). To avoid these problems, a so-called deep eutectic solvent (DES) can be used.

The principles of DES were firstly described by Abbott et al. (2002). Generally, a DES consists of an ammonium or phosphonium salt and a hydrogen-bond donor, e.g., an amine, amide, alcohol, or carboxylic acid (Abbott et al. 2004). By mixing these components the hydrogen-bond donor interacts with the anion of the salt, followed by a drastic reduction of the solution's melting point (Durand et al. 2012; Tang and Row 2013).

Recent publications demonstrate the successful application of DES as a potential solvent for the production of different chemicals like polymers, biodiesel, or for several additional, cyclization, or condensation reactions (Carriazo et al. 2012; Kumar and Sharma 2017; Zhang et al. 2012; Zhao et al. 2013). Furthermore, DES can act as solvent and substrate for the enzymatic synthesis of glycolipids at the same time using choline chloride as ammonium salt and a sugar as hydrogen-bond donor, making the usage of harmful organic solvents unnecessary (Siebenhaller et al. 2017).

The current paper focuses on a proof of principle of the sustainable lipase-catalyzed synthesis of glycolipids in a DES consisting of choline chloride and a purified and dried beechwood cellulose hydrolysate. Here, the pretreatment of the monosaccharide solution, synthesis, purification, determination of the glycolipid yields and molecular weight, as well as structural elucidation of the resulting glycolipids is described.

## Methods

### Materials

Lipase B from *Candida antarctica*, immobilized on acrylic resin (former Novozyme 435) and choline chloride (98%) were purchased from Sigma-Aldrich (Sigma-Aldrich, St. Louis, USA). CDCl_3_ (99.8%) and acetone-d6 (>99.9%) were from Eurisotop (Gif-sur-Yvette Cedex, France). Activated carbon was acquired from Carl Roth (Carl Roth KG, Karlsruhe, Germany). Fatty acids were purchased from Tokyo Chemical Industry Co. Ltd (TCI-Europe, Zwijndrecht, Belgium). All solvents were purchased in the highest purity available and used without further treatment.

### Preparation of the monosaccharide solution

The monosaccharide solution applied in the investigations was obtained by beechwood fractionation via acid-catalyzed organosolv process. Enzymatic hydrolysis of the fiber and the evaporation of water resulted in the following mono- and di-saccharide concentrations in the hydrolysate: 31 g/l cellobiose, 90 g/l xylose, and 608 g/l glucose. The samples were diluted to obtain suitable concentrations (<10 g/l) for HPLC measurement (Ludwig et al. 2013). The chromatographic analysis was performed using an Aminex HPX-87H column (Bio-Rad Laboratories, Germany) as stationary phase and 5 mM sulfuric acid as mobile phase. Separation at 30 °C and a volumetric flow of 0.6 ml/min enabled the identification of single compounds with a refractive index detector (8120, Bischoff Chromatography, Germany). Common degradation products such as HMF, furfural, or acetic acids which can also be quantified by the application of the HPLC method were either not detected or below the limit of determination. Calibration with pure standards ranged from 0.5 to 10 g/l.

### Pretreatment of the monosaccharide solution

The monosaccharide solution was further purified to remove other interfering substances like phenolic or lignin compounds which are not detectable with the HPLC method described before. For this, the fraction was diluted in ddH_2_O (1:2 w/v) and 1 g activated carbon per 5 ml solution was added. After rigorous shaking for 1 min, the activated carbon sugar mixture was incubated for 3 h at room temperature. Succeeding purification, the activated carbon was separated by a two-step filtration process (pore size 4–7 and 0.22 µm). Afterwards, the sugar solution was spray dried in a werco^®^ SD-20 spray dryer (input temperature 182 °C, output temperature 74–81 °C; Hans G. Werner Industrietechnik, Reutlingen, Germany). The sugar mix had a final water content of 8% (TitroLine^®^ 7500 KF trace, SI Analytics, Mainz, Germany).

The two main carbohydrates, glucose and xylose, were quantified after drying by HPLC (Agilent 1100 Series, Agilent Technology, Waldbronn, Germany) as described in Buchholz et al. (2013) with slight modifications. The flow rate was set to 0.5 ml/min and the temperature of the refractive index detector (Agilent 1200 Series) was set to 50 °C, too.

### Preparation of deep eutectic solvents

DES for the synthesis for glycolipids from beechwood carbohydrates were prepared by mixing choline chloride with the purified and dried sugar mix at a given ratio of 1:1.3 (w/w). For the production of glucose– and xylose–octanoates as standards, CC and the corresponding sugar were mixed in a molar ratio of 1:1. The mixtures were constantly stirred at 100 °C until a liquid DES was formed.

### Synthesis and extraction of glycolipids

The synthesis reactions were performed by adding 100 mg of lipase Novozyme 435 and 1.5 mmol of the fatty acid (vinyl-octanoate or octanoic acid) to 3.5 ml of the formed DES in a 5 ml Eppendorf cup. The synthesis reaction was incubated for 72 h at 50 °C in a rotator with vortex mixer in program U2 at 50 rpm (neoLab, Heidelberg, Germany). Negative controls were carried out without lipase or fatty acid.

After 72 h the DES was dissolved while shaking by adding 1 ml of 70 °C hot ddH_2_O until full dissolution of the DES was reached. To perform glycolipid extraction, the solved DES solution was added in a ratio of 1:1 (by vol.) to ethyl acetate and the mixture was shaken for 45 s. Afterwards, the glycolipid-containing organic phase was analyzed via thin-layer chromatography.

In order to determine the yields of the enzymatic reaction, it is essential to extract all synthesized glycolipids from the DES. To accomplish that, the solved DES solution was extracted 5 times with ethyl acetate (1:1, by vol.). These extracts were merged and used for the LC–MS/MS analysis.

### Quantitative detection of glycolipids via thin-layer chromatography

For quantitative analysis of glycolipids, 10 µl of extracted samples was spotted onto a silica gel TLC plate (Alugram SIL G, 60 Å, Macherey–Nagel GmbH & Co.KG, Düren, Germany). The separation of synthesized compounds were performed with a mobile phase consisting of chloroform:methanol:acetic acid (65:15:2 or 35:1:1, by vol.). Visualization was accomplished by incubation of the TLC plate in a staining solution (anisaldehyde:sulfuric acid:acetic acid 0.5:1:100, by vol.) followed by heating under a 200 °C continuous air flow for up to 5 min.

### Purification and fractionation of glycolipid extracts by flash chromatography

The glycolipid extracts were purified by flash chromatography previous to further analysis with mass spectrometry or NMR. For this purpose, multiple extracts from 12 identical synthesis reactions were combined and concentrated to 4 ml (50 mbar, 50 °C, and 2.000 rpm; Heidolph Laborota 4000, Schwabach, Germany). The compounds of the concentrated solution were separated by employing a hydrophobic silica column with a SepacoreX50 (40–63 µm particle size, 60 Å pore size, 150 mm column length, 12 mm column diameter; Büchi Labortechnik GmbH, Flawil, Switzerland). A gradient of chloroform:hexane (4:1, by vol.) and methanol were used as solvent at a constant flow rate of 5 ml/min. Elutes were collected in 5 ml fractions and analyzed via TLC.

The synthesized glucose– and xylose–octanoates to be used as LC–MS/MS standards were purified applying a Reveleris^®^ Prep System with a Reveleris^®^ Silica 12 g column (Büchi Labortechnik GmbH, Flawil, Switzerland). A gradient of chloroform (A) and methanol (B) was used (in 2 min from 0 to 5% B, increase the gradient gradually in 10 min to 10%, then in 1 min to 100% B and keep it constant for 2 min). Peaks were detected by an ELSD detector (Threshold 25 mV). The glucose–octanoate respectively xylose–octanoate fractions were collected, concentrated, and transferred to pre-weighed round-bottom flasks. The eluent was completely evaporated in a vacuum concentrator (50 mbar, 50 °C, and 2.000 rpm; Heidolph Laborota 4000, Schwabach, Germany). The flasks were weighed again to determine the amount of pure sugar esters to be used as standards during LC–MS/MS.

### Determination of the accurate masses and structure via electrospray ionization quadrupole time-of-flight mass spectrometry (ESI–Q–ToF MS)

An ESI–Q–ToF MS system (Q-Star Pulsar i, AB SCIEX, Darmstadt, Germany) equipped with an electrospray ionization (ESI) source was used for mass determination of the purified compounds. Measurements were carried out in the positive mode within a mass range from *m/z* 50 to *m/z* 800 using the activated “enhance all” setting. Samples were diluted 1:5 in a mixture of methanol and 10 mM ammonium acetate (1:1, by vol.) and continuously infused via a syringe pump at a flow rate of 10 µl/min.

The ion source voltage was set at 4800 V, declustering potential was adjusted at 30 V and focusing potential at 100 V. In all experiments nitrogen gas 5.0 was used as nebulizer and curtain gas.

Data acquisition and processing were performed using the Analyst QS 1.1 software (AB SCIEX, Darmstadt, Germany).

### LC–MS/MS analyses for glycolipid quantification

Mass spectrometric analyses were done using an API 4000™ quadrupole mass spectrometer (Applied Biosystems/MDS Sciex Toronto, Canada) equipped with an electrospray ionization (ESI) source. MS spectra were generated by infusion experiments using a syringe pump (Harvard Apparatus Inc. South Natick, MA, USA). Single MS experiments (Q1 scan) and MS/MS experiments (product ion scan, PIS) were used to get structural information. Nitrogen 5.0 was used as curtain gas, nebulizer gas, and collision gas.

The purified standard compounds, glucose–octanoate and xylose–octanoate, were diluted in a solvent mixture of acetonitrile/10 mM ammonium acetate (50:50, v/v) and infused with a flow rate of 0.80 ml/h. MS experiments were carried out in the positive ionization mode using an ion spray voltage of 4800 V, a declustering potential of 30 V, and an entrance potential of 10 V. MS/MS experiments were generated using the compound optimization mode in the software Analyst V 1.6.

For both targets three mass transitions (one quantifier and two qualifiers) were selected (Table 1).

**Table 1:** Targets of the LC–MS/MS analyses with the corresponding mass transition of the quantifier and both qualifiers

Target	*m*/*z* Quantifier	*m*/*z* Qualifier 1	*m*/*z* Qualifier 2
Glucose–octanoate	307/289	307/271	307/127
Xylose–octanoate	277/259	277/127	277/115

A calibration function from 100 to 1000 µg/l for each sugar ester was prepared for quantification of the extracted targets (see Additional file 1).

An Agilent 1100 HPLC system (Agilent Waldbronn, Germany) was used for sample separation on a Multospher 120 AQ RP C-18, 5 µm column (250 × 4 mm). A gradient of acetonitrile (A) and 10 mM ammonium acetate (B) were used by a total run time of 25 min (start with 2 min of 30% A, increase it in 3 min to 60%, and hold it for 7 min. Afterwards switch in 1 min back to 30% and hold it for 12 min).

The ion source temperature was set to 400 °C and a flow rate of 500 µl/min was applied. The injection volume for all samples was 40 µl.

### Structural elucidation via nuclear magnetic resonance spectroscopy

For NMR spectroscopy, 3.9 mg of purified fractions 105–106 (with octanoic acid) from the flash chromatography was dissolved in 0.6 ml CDCl_3_/d6-acetone (4:1, by vol.). 1D ^1^H NMR spectroscopy and 2D ^1^H–^1^H correlation spectroscopy (COSY), ^1^H–^13^C heteronuclear single quantum coherence spectroscopy [clip-HSQC (Enthart et al. 2008)], and heteronuclear multiple-bond correlation spectroscopy (HMBC) were recorded on a Bruker AVANCE II +600-MHz spectrometer (Bruker AG, Rheinstetten, Germany) equipped with a BBI probe head. Spectra were analyzed with Topspin 3.2 (Bruker AG) and SpinWorks 3.1.8 software (Marat, University of Manitoba). Intensities were measured from a 1D ^1^H spectrum acquired with a sixteen scan and four dummy scans. Chemical shifts are referenced to the ^1^H and ^13^C resonance of tetramethylsilan.

## Results and discussion

### Properties of the DES reaction media

The purified and dried sugar mix consists of 71.6% glucose and 16.6% xylose; cellobiose could not be detected via HPLC. With this data, it was calculated that each reaction approach with 3.5 ml DES contains 1.81 g glucose (10.06 mmol) and 0.42 g (0.002 mmol) xylose.

The prepared DES had a final water content of 5.4% and a pH of 7.7 (SenTix^®^ Mic, Xylem Analytics, Weilheim, Germany), which fits in the optimum pH range for the used lipase. Hayyan et al. investigated the physical properties of different glucose-based DES and concluded that this kind of DES has a nearly neutral pH because of the low acidity of d-glucose, which made them an ideal media for biological and chemical applications (Hayyan et al. 2013).

After a reaction time of 72 h, the DES had a pH of about 5.7, whereas the pH control of pure DES and of a DES with 1.5 mmol vinyl-octanoate dropped to approx. pH 6.6.

### Glycolipid detection via thin-layer chromatography

To confirm the successful enzymatic formation of sugar esters the extracts were analyzed via TLC. In negative controls without lipase or fatty acid two slightly spots were visible. These spots are identical with the standards of pure glucose (Rf 0.1) and xylose (Rf 0.16) which demonstrated that these spots are sugars of the sugar mix in the used DES.

**Fig. 1:**
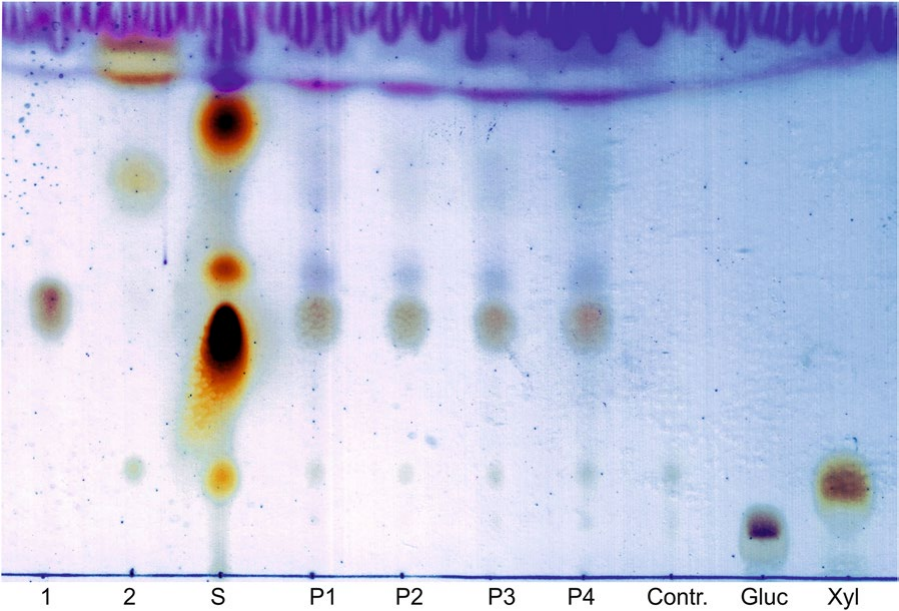
The TLC shows the glycolipid standards glucose–octanoate (*1*) and xylose–octanoate (*2*), synthesized with vinyl-octanoate. *S* is a lab-intern rhamnolipid standard, which acts as a positive control for the TLC. *P1*–*P4* shows extracts of the reaction in a CC:sugar mix DES with vinyl-octanoate as substrate. At a Rf between 0.1 and 0.2 there is mainly the sugars, glucose and xylose, out of the DES, which are comparable with the pure sugar standards of glucose (*Gluc*) and xylose (*Xyl*). The brownish double spots and the purple spots (*P1*–*P4*) represent different glucose–octanoates (Rf 0.4–0.5) and the slightly spot with a Rf of 0.65–0.75 xylose–octanoate. The *spot on the height of the running front* indicates more non-polar products, like di- or poly-acylated sugars. A negative control without enzyme and without lipase shows only glucose and xylose spots (*Contr*.)

**Fig. 2:**
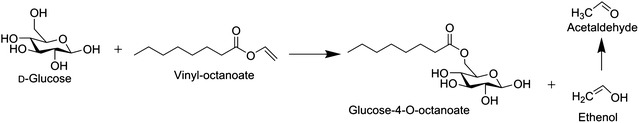
Transesterification between glucose and vinyl-octanoate which leads to the formation of glucose–4–*O*–octanoate and ethenol. Ethenol is not stable and tautomerizes to acetaldehyde. Acetaldehyde evaporates quickly, pushing the reaction forward. The reaction scheme is analogous for other monosaccharides like xylose. It might be possible that more or other C-atoms are acylated, too. Using a fatty acid like octanoic acid, one molecule of water will be formed as a side product

The visualized extracts of the synthesis reaction with vinyl-octanoate as substrate shows, besides the sugar spots, four more spots (Fig. 1). These new spots are potential glucose–octanoate and xylose–octanoate spots with different conformations. Furthermore, the spots with Rf 1 indicate the formation of small amounts of highly non-polar sugar-di- or poly-octanoates (Fig. 2). This leads to the assumption that the used lipase is not specific for mono-acylated products under the given conditions and can form traces of di- or poly-acylated sugars and not a defined single product. This result agrees with those of earlier investigations (Siebenhaller et al. 2017).

Moreover, the transesterification with vinyl-octanoate additionally releases ethenol, which tautomerizes to a highly volatile acetaldehyde. This should push the reaction forwards and can help to accelerate the reaction (Fig. 2). On the other hand, under the nearly water-free conditions (approx. 5.4% water), it can result in the formation of sugar aldehydes if the acetaldehyde does not evaporate fast enough. To counteract this, a synthesis reaction under reduced pressure might be a possible option.

Further synthesis reactions were made with octanoic acid as substrate, leading to a similar spot pattern (data not shown). Using this substrate, glucose– and xylose–octanoate will be formed, too.

Since only qualitative results are obtained using TLC, samples which possibly contain glycolipids have been characterized further by mass determination and nuclear magnetic resonance spectroscopy.

### Mass determination via electrospray ionization quadrupole time-of-flight mass spectrometry (ESI–Q–ToF MS)

For validation of the successful enzymatic synthesis of glycolipids with octanoic acid as substrate, appropriate masses of glucose–octanoate (M_G_) were detected via ESI–Q–ToF MS in combined fraction 105 + 106 of the flash chromatography (see Q1 mass spectra in the Additional file 2). The presence of ions at *m*/*z* 271.134 (M_G_–2H_2_O), *m*/*z* 289.148 (M_G_–H_2_O), and the sodium adduct at *m*/*z* 329.150 (M_G_ + Na^+^) verify the assumed reaction (Fig. 2).

Using vinyl-octanoate as substrate, the masses in combined fraction 26 + 27 were measured, too. The proof for the correct formation of glucose–octanoate is clearly elucidated by the presence of the ion at *m*/*z* 271.150 (M_G_–2 H_2_O). Furthermore, the existence of the sodium adduct of glucose-di-octanoate (*m*/*z* 455.261) confirms the formation of sugar-di-octanoates. All acquired *m*/*z* values of both samples are listed in Table 2.

**Table 2:** Observed *m*/*z* values during ESI–Q–ToF experiment of samples 26 + 27 with vinyl-octanoate and 105 + 106 with octanoic acid are shown

Observed *m*/*z* value	Calculated *m*/*z* value	Sample	Corresponding fragment
271.134	271.146	105 + 106 and 26 + 27	M_G_−2H_2_O
289.148	289.157	105 + 106	M_G_−H_2_O
329.150	329.157	105 + 106	M_G_ + Na^+^
415.262	415.262	26 + 27	M_G2_−H_2_O
455.264	455.262	26 + 27	M_G2_ + Na^+^

Masses compared to glucose–octanoate with a calculated molar mass of 306.168 Da (=M_G_) or to glucose-di-octanoate of 432.272 Da (=M_G2_)

The formation of glycolipids by Novozyme 435 in a DES, consisting of CC and a purified fiber hydrolysate, was thus successful. The proof of the existence of the sodium adducts of glucose–octanoate (*m*/*z* 329.150) and glucose-di-octanoate (*m*/*z* 455.261) confirms the proposed reaction.

Strangely in this two tested fractions no xylose–octanoates (M_X_) were detected, although xylose is present in small amounts in the fiber hydrolysate, respectively in the sugar mix.

Even so, the presence of xylose–octanoates could be demonstrated with the used substrates in other measured samples. The presence of the sodium adduct of xylose–octanoate was identified at *m*/*z* 299.132 in fraction 99 (octanoic acid), in fraction 33–36 the fragment M_X_–H_2_O was observed at *m*/*z* 259.148 (vinyl-octanoate). Since the used pretreated hydrolysate for all DES was the same, xylose–octanoates should be formed in low amounts in every reaction.

For complete elucidation of the correct chemical structure of the obtained glucose– and xylose–octanoate fraction, 105 + 106 was analyzed using NMR.

### Determination of the synthesis yields by LC–MS/MS analysis

Glycolipids from four synthesis reactions with vinyl-octanoate, based on a CC:sugar mix DES, were separately extracted and analyzed by LC–MS/MS. On average, 0.069 mmol of glycolipid (21.4 mg) was formed in one synthesis reaction, of which the main product, with >97%, is glucose–octanoate (Table 3). Only small amounts of xylose–octanoate were formed, as a consequence of glucose being 4 times more abundant than xylose (see Additional file 3). Furthermore, glucose seems to be favored by the enzyme, which becomes clear when calculating conversion yields for glucose and xylose.

**Table 3:** Measured amounts of synthesized glucose–octanoate and xylose–octanoate in one reaction setup with 3.5 ml of DES, 100 mg Novozyme 435, and 1.5 mmol vinyl-octanoate

Reaction	Yield (%)	Total amount (mg)	Glucose–octanoate (mg)	Xylose–octanoate (mg)
P1	5.1	22.7	22.1	0.67
P2	4.9	21.7	21.1	0.62
P3	4.6	20.6	20.0	0.64
P4	4.6	20.5	19.9	0.61

The total amount of approx. 0.07 mmol synthesized glycolipid correspond to a yield of 4.81%

With this setup, a maximum of 1.5 mmol glycolipids could be synthesized, being this limited by the amount of used octanoic acid. Thus, the overall reaction conversion yield is 4.81%.

On the other hand, roughly 0.7% of the glucose in one approach is converted to glucose–octanoate, whereas less than 0.1% of xylose was used to form xylose–octanoate.

Synthesized sugar-di-octanoates were not included in the calculation of the yield due to their low occurrence.

Being this is a non-optimized synthesis setup, it was still possible to produce small amounts of different glycolipids from beechwood carbohydrates. When compared to other enzymatic glycolipid synthesis reactions in organic solvents the achieved yields are low. Depending on the solvent, enzyme, substrates and time, as well as the addition of molecular sieves, it is possible to convert 100% of the sugar to a glycolipid (Amos et al. 1998), but in other systems the yield is lower (Ducret et al. 1996; Tarahomjoo and Alemzadeh 2003).

To achieve higher yields, the process can be improved by optimizing various parameters. One such parameter is the water content of the DES. It has already been shown that enzymatic reactions can be accelerated by increasing the water content of the DES, so it is important to find the optimal water concentration (Durand et al. 2013; Guajardo et al. 2017). Furthermore, it should be possible to set the pH to the lipase optimum by adding a buffer to the DES and not just pure water. Another parameter to improve is the amount of the used enzyme, as well as the fatty acid, since high concentrations can have negative effects on the enzyme. The adjusted temperature of 50 °C is in the optimum range of the lipase, but it is also stable and active at slightly higher temperatures. Increasing the temperature a few degrees would decrease the viscosity (Hayyan et al. 2013), which leads to a better mass transport. Moreover, the mixing of the synthesis reaction has to be improved to create a more homogenous reaction mixture, which will improve the yields.

### Configuration of sugar-octanoates structure by NMR

The sample 105 + 106 was analyzed with ^1^H COSY, ^13^C HSQC, and ^1^H^13^C HMBC spectra. Two major carbohydrate systems were identified and studied, starting from the anomeric protons of the ^1^H COSY and ^13^C HMBC spectra. Both systems are C6 sugars which were identified as α- and β-glucose. The glucose moieties are acylated with octanoic acid at the C6 atoms, confirmed on the basis of cross peaks of protons with lipid carbonyls in the ^1^H^13^C HMBC (Table 4).

**Table 4:** Chemical shifts of the main products present in fraction 105 + 106

	C shift (ppm)	H shift (ppm)	Multiplicity	Coupling (Hz)
α-glucose				
─C^1^H─O─	92.50	5.27	d	2.83
─C^2^H─	72.45	3.53	t	9.40
─C^3^H─	73.97	3.82	t	9.40
─C^4^H─	70.18	3.41	t	9.40
─C^5^H─	69.56	4.03	d	9.40
─C^6^H─ (acylated C′173.93)*	63.20	4.30	Overlap	nd
─C^6′^H─ (acylated C′173.93)*	63.20	4.40	Overlap	nd
β-glucose				
─C^1^H─O─	96.87	4.64	d	6.40
─C^2^H─	74.88	3.35	t	8.60
─C^3^H─	76.37	3.55	t	8.80
─C^4^H─	70.18	3.46	Overlap	nd
─C^5^H─	74.03	3.54	Overlap	nd
─C^6^H─ (acylated C′174.02)*	63.40	4.32	Overlap	nd
─C^6′^H─ (acylated C′174.02)*	63.40	4.37	Overlap	nd

*d* doublet, *t* triplet, *nd* not determinable

* Acylation site

Furthermore, sample 101 + 102 was analyzed (Additional file 4). In this sample two major carbohydrate systems were identified. These systems are β-glucoses which were acylated with the fatty acid at the C6, too.

In addition to the main systems there are minor systems presented with many overlaps. One of these minor systems could be identified as the pentose β-xylose. This β-xylose is acylated at the OH-group at the C4.

Compared to the two glucose systems, the β-xylose system is acylated on the OH-group at the C4 with octanoic acid. The detection of xylose–octanoate in this fraction was expected, based on the results of the ESI–Q–ToF MS measurements.

These NMR data confirm the results of the previous mass spectrometry. The synthesis of glucose-6-octanoate and xylose-5-octanoate in a DES consisting of CC and sugars from a lignocellulose hydrolysate with Novozyme 435 was successfully proven.

From the collected data it can be speculated so far that different sugar molecules have different preferred acylation sites. In previous work, arabinose, a pentose, was used as sugar for the synthesis of glycolipids, and vinyl laurate served as fatty acid component (Siebenhaller et al. 2017). Two acylated arabinose systems were detected in NMR measurements. One system was acylated at the C3 and the other at C4, or one system was di-acylated at C3 and C4 while the other system was pure arabinose.

The C4 hydroxy group is maybe favored at pentoses like arabinose and xylose, but the other hydroxyl groups are potential acylation targets, too. In glucose, a hexose, the C6 OH-group seems to be favored, because this group has less steric effects due to its position and offers a good access for the lipase active site.

## Conclusions

Albeit having used a non-optimized process, a first step to the production of sustainable glycolipids was achieved. The sugars used for the enzymatic synthesis of sugar esters were extracted from beechwood lignocellulose, a renewable and frequently available resource. By the formation of a DES, the sugars contained are easily available in the reaction solvent. This is an elegant way to avoid the negative effect of the low solubility of sugars in other water-free solvents. The successful formation of different glucose– and xylose–octanoates was confirmed by ESI–ToF mass spectrometry and multidimensional NMR measurements. With the used reaction setup it was possible to achieve a yield of 4.81% of the maximum yield, but there is great potential to further optimize the system, by addressing various parameters.

## Additional files

**Additional file 1.** Results of the LC-MS/MS calibration with glucose-octanoate and xylose-octanoate.

**Additional file 2.** Raw data of the ESI-Q-ToF measurements. “PO” indicates a synthesis reaction with octanoic acid; “PV” with vinyl octanoate. “Blank” is a mixture of methanol and 10 mM ammonium acetate (1 : 1, by vol.).

**Additional file 3.** Resulting yields of glucose-octanoate and xylose-octanoate performed by LC-MS/MS measurement.

**Additional file 4.** Chemical shifts of fraction 101 + 102 of glucose and xylose.

## Abbreviations

DES: deep eutectic solvent(s)
CC: choline chloride
Rf: retention factor
