# Supplementary figures and images for: Beechwood carbohydrates for enzymatic synthesis of sustainable glycolipids
Source: Bioresour Bioprocess. 2017 Jun 7;4(1):25. doi: 10.1186/s40643-017-0155-7 (PMC5487819; doi:10.1186/s40643-017-0155-7)

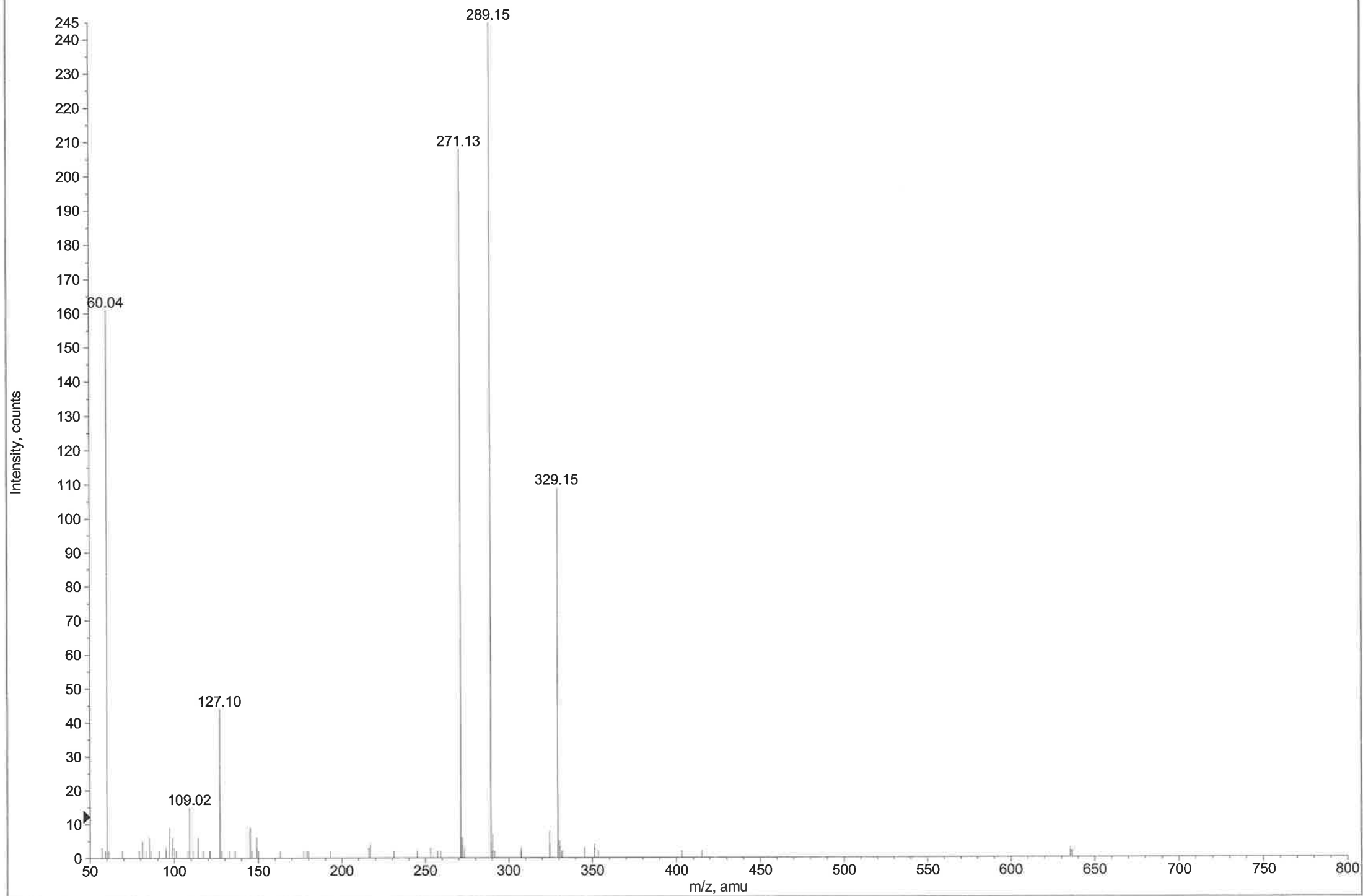

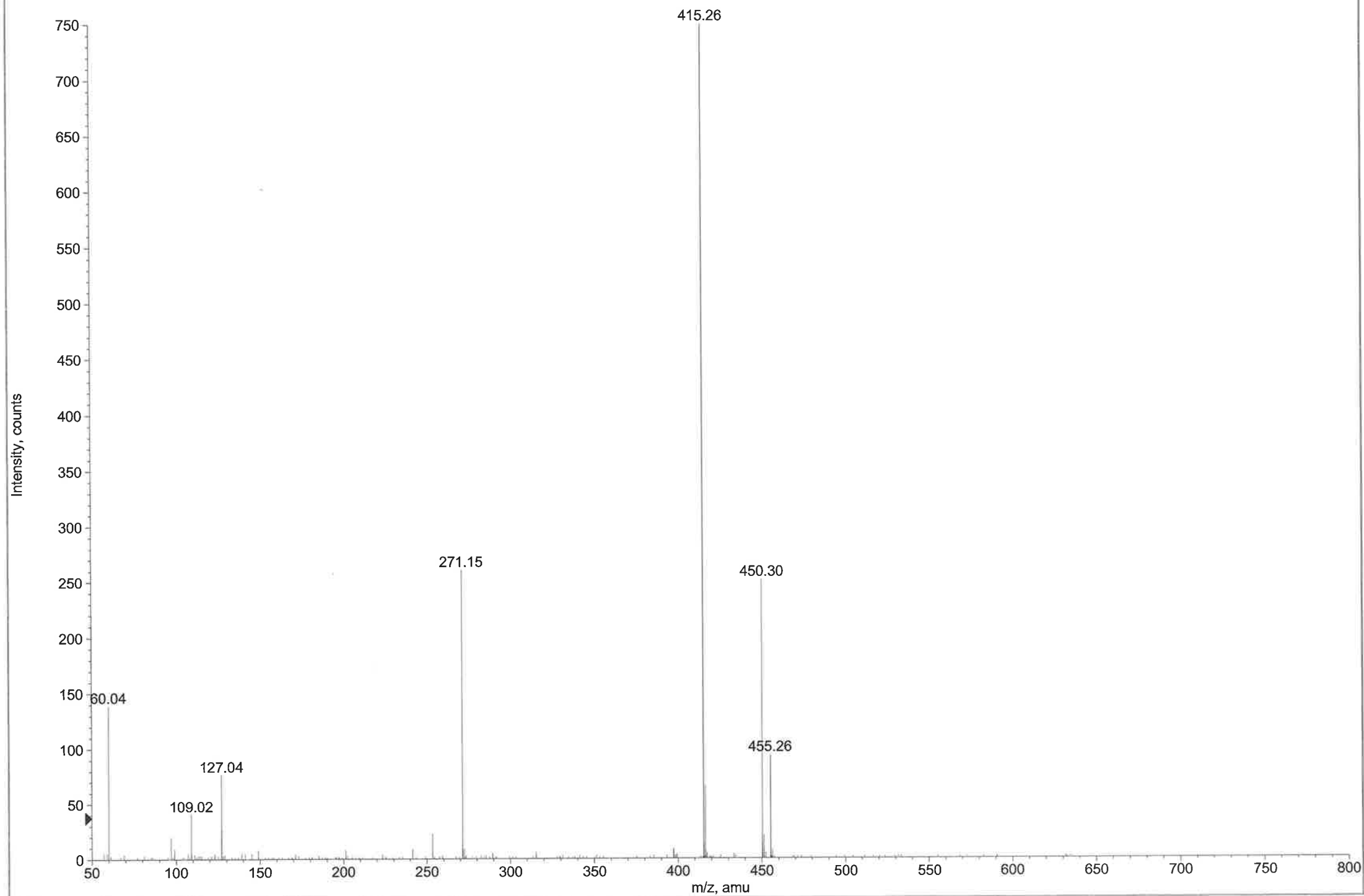

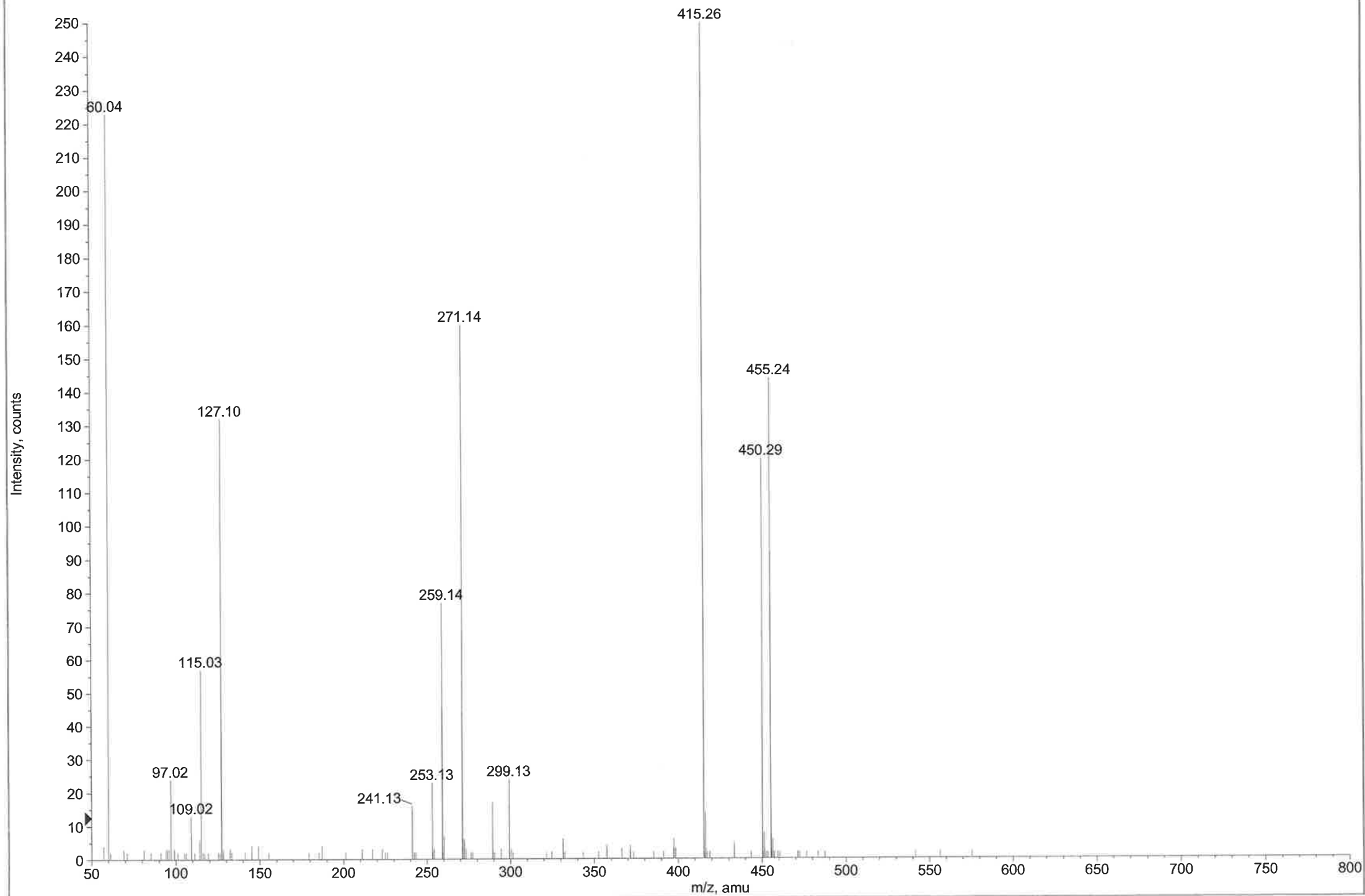

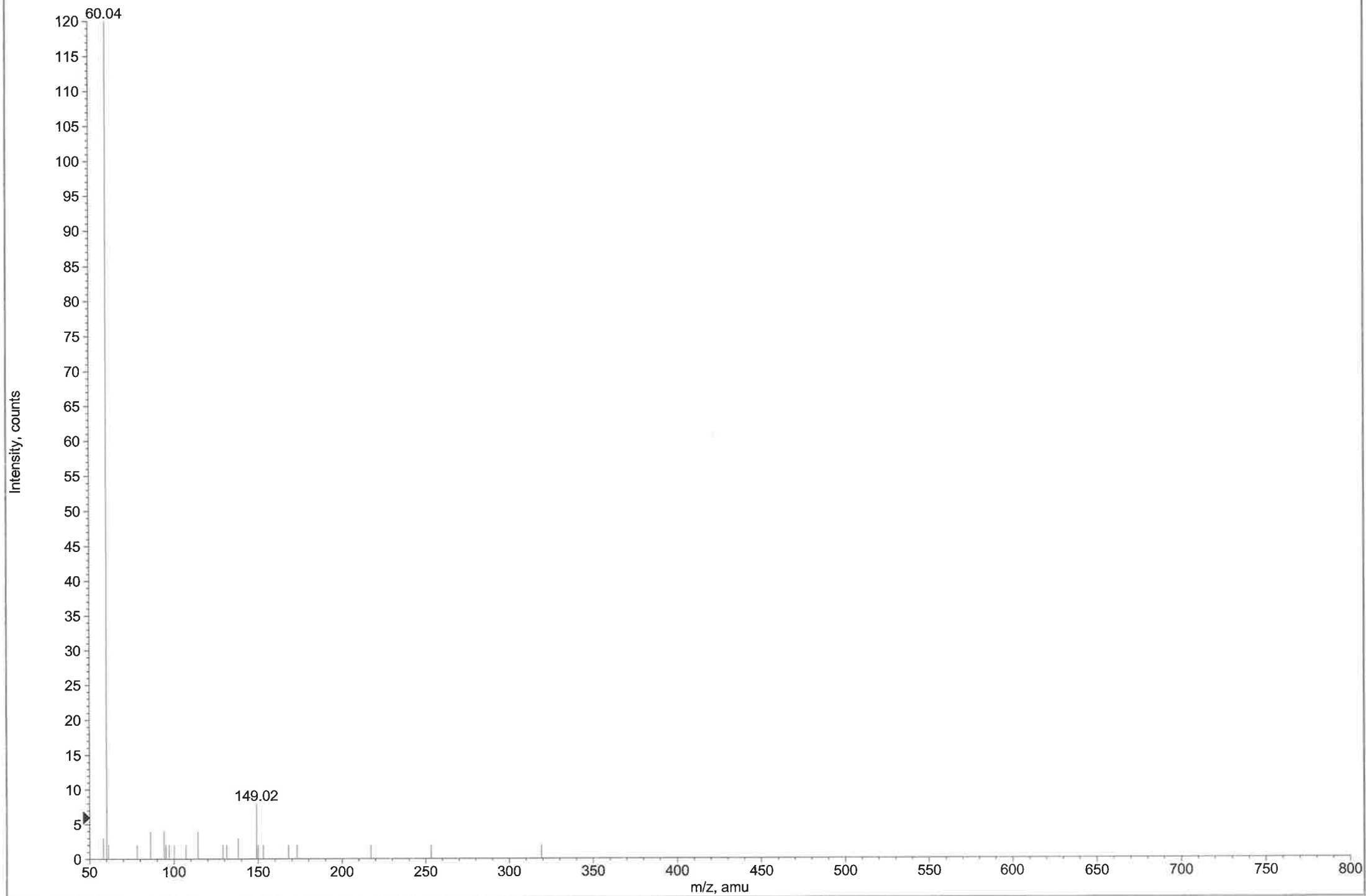

Supplement: Supplementary file 2 — Additional file 2. Raw data of the ESI-Q-ToF measurements. “PO” indicates a synthesis reaction with octanoic acid; “PV” with vinyl octanoate. “Blank” is a mixture of methanol and 10 mM ammonium acetate (1 : 1, by vol.). [file 40643_2017_155_MOESM2_ESM.pdf]
