# Supplementary material for: Beechwood carbohydrates for enzymatic synthesis of sustainable glycolipids
Source: Bioresour Bioprocess. 2017 Jun 7;4(1):25. doi: 10.1186/s40643-017-0155-7 (PMC5487819; doi:10.1186/s40643-017-0155-7)
Supplement: Supplementary file 3 — Additional file 3. Resulting yields of glucose-octanoate and xylose-octanoate performed by LC-MS/MS measurement. [file 40643_2017_155_MOESM3_ESM.pdf]

Results LC-MS/MS with xylose-octanaote

Script Help

Quantitate Mode

Sascha_Octanoate\Octanoate_

Full Layout
Query: None
Idle
Sort : Unsorted

	Sample Name	Sample Type	Acquisition Method	File Name	Analyte Peak Area (counts)	Analyte Peak Height (cps)	Analyte Concentration (ng/mL)	Analyte Retention Time (min)	Calculated Concentration (ng/mL)	Accuracy (%)
1	Blank L14_01_08	Standard	Octanoate_MRM_POS_TI	Cal_Octanoate_15-	2.04e+002	3.87e+001	0.00	12.6	6.04	N/A
2	STD_Xyloc_100ng in L14_0	Standard	Octanoate_MRM_POS_TI	Cal_Octanoate_15-	1.10e+004	1.24e+003	100.	12.4	102.	102.
3	STD_Xyloc_200ng in L14_0	Standard	Octanoate_MRM_POS_TI	Cal_Octanoate_15-	2.24e+004	2.56e+003	200.	12.6	204.	102.
4	STD_Xyloc_500ng in L14_0	Standard	Octanoate_MRM_POS_TI	Cal_Octanoate_15-	5.32e+004	5.62e+003	500.	12.6	478.	95.6
5	STD_Xyloc_1000ng in L14_	Standard	Octanoate_MRM_POS_TI	Cal_Octanoate_15-	1.13e+005	1.31e+004	1000.	12.4	1010.	101.
6	P2_1_200_(10mL) L14_01_	Unknown	Octanoate_MRM_POS_TI	Sascha_Proben_17	1.36e+005	9.45e+003	N/A	12.5	1220.	N/A
7	P3_1_200_(10mL) L14_01_	Unknown	Octanoate_MRM_POS_TI	Sascha_Proben_17	1.42e+005	1.01e+004	N/A	12.3	1270.	N/A
8	P4_1_200_(10mL) L14_01_	Unknown	Octanoate_MRM_POS_TI	Sascha_Proben_17	1.39e+005	9.99e+003	N/A	12.5	1240.	N/A
9	P5_1_200_(10mL) L14_01_	Unknown	Octanoate_MRM_POS_TI	Sascha_Proben_17	1.49e+005	1.17e+004	N/A	12.3	1330.	N/A

Results LC-MS/MS with glucose-octanaote

Script Help

Quantitate Mode

Sascha_Octanoate\Octanoate_

Full Layout
Query: None
Idle
Sort : Unsorted

	Sample Name	Sample Type	Acquisition Method	File Name	Analyte Peak Area (counts)	Analyte Peak Height (cps)	Analyte Concentration (ng/mL)	Analyte Retention Time (min)	Calculated Concentration (ng/mL)	Accuracy (%)
1	Blank L14_01_08	Standard	Octanoate_MRM_POS_TIS.dam	Cal_Octanoate_15-	1.50e+002	3.09e+001	0.00	11.2	17.3	N/A
2	STD_Gluc_100ng in L14_01_04	Standard	Octanoate_MRM_POS_TIS.dam	Cal_Octanoate_15-	6.70e+003	9.28e+002	100.	11.1	98.3	98.3
3	STD_Gluc_200ng in L14_01_05	Standard	Octanoate_MRM_POS_TIS.dam	Cal_Octanoate_15-	1.50e+004	1.91e+003	200.	11.1	202.	101.
4	STD_Gluc_500ng in L14_01_06	Standard	Octanoate_MRM_POS_TIS.dam	Cal_Octanoate_15-	3.64e+004	4.66e+003	500.	11.2	466.	93.2
5	STD_Gluc_1000ng in L14_01_07	Standard	Octanoate_MRM_POS_TIS.dam	Cal_Octanoate_15-	8.09e+004	1.12e+004	1000.	11.2	1020.	102.
6	P2_1_20000_(10mL) L14_01_05	Unknown	Octanoate_MRM_POS_TIS.dam	Sascha_Proben_17	3.09e+004	3.42e+003	N/A	11.1	398.	N/A
7	P3_1_20000_(10mL) L14_01_07	Unknown	Octanoate_MRM_POS_TIS.dam	Sascha_Proben_17	3.10e+004	3.88e+003	N/A	11.2	399.	N/A
8	P4_1_20000_(10mL) L14_01_09	Unknown	Octanoate_MRM_POS_TIS.dam	Sascha_Proben_17	3.28e+004	3.72e+003	N/A	11.2	422.	N/A
9	P5_1_20000_(10mL) L14_01_11	Unknown	Octanoate_MRM_POS_TIS.dam	Sascha_Proben_17	3.45e+004	4.06e+003	N/A	11.1	442.	N/A
