# Supplementary material for: Beechwood carbohydrates for enzymatic synthesis of sustainable glycolipids
Source: Bioresour Bioprocess. 2017 Jun 7;4(1):25. doi: 10.1186/s40643-017-0155-7 (PMC5487819; doi:10.1186/s40643-017-0155-7)
Supplement: Supplementary file 4 — Additional file 4. Chemical shifts of fraction 101 + 102 of glucose and xylose. [file 40643_2017_155_MOESM4_ESM.pdf]

β-Glucose	C Shift [ppm]	H Shift [ppm]	Multiplicity	Coupling [Hz]
**–C^{1}H–**O–	92.41	5.26	d	7.2
**–C^{2}H–**	73.87	3.52	Overlap	n.d.
**–C^{3}H–**	73.94	3.82	Overlap	n.d.
**–C^{4}H–**	70.18	3.42	t	6.8
**–C^{5}H–**	69.53	4.03	dd	7.6
**–C^{6}H- (*acylated C`173.56)**	63.29	4.30	dd	5.3
**–C$^{6'}$H- (*acylated C`173.56)**	63.29	4.32	dd	5.5

β-Glucose	C Shift [ppm]	H Shift [ppm]	Multiplicity	Coupling [Hz]
**–C^{1}H–**O–	96.97	4.63	d	7.02
**–C^{2}H–**	73.61	3.34	t	7.8
**–C^{3}H–**	76.42	3.55	Overlap	n.d.
**–C^{4}H–**	70.19	3.45	Overlap	n.d.
**–C^{5}H–**	76.41	3.54	t	6.7
**–C^{6}H- (*acylated C`173.91)**	63.29	4.38	dd	4.84
**–C$^{6'}$H- (*acylated C`173.91)**	63.29	4.4	dd	4.84

β-Xylose	C Shift [ppm]	H Shift [ppm]	Multiplicity	Coupling [Hz]
**–C^{1}H-**	79.63	5.21	t	7.8
**–C^{2}H–**	72.65	3.56	Overlap	n.d.
**–C^{3}H–**	71.23	3.95	t	8.7
**–C^{4}H– (*acylated C`173.08)**	71.36	4.79	ddd	9.1
**–C^{5}H-**	58.95	3.75	d	5.36
**–C$^{5'}$H-**	58.95	3.73	d	5.8
